# Family Caregivers’ Perspectives on Challenges and Support Needs in Hospital-Based Palliative Care for Persons Living With Dementia

**DOI:** 10.1177/07334648251389300

**Published:** 2025-11-17

**Authors:** Jung Kwak, Anita Chary, Sarah Stayer, Kwaku Duah Oppong, Sumin Yoon, Snehal Patel, Elizabeth A. Kvale

**Affiliations:** 1 School of Nursing, The University of Texas at Austin, Austin, TX, USA; 2 Effectiveness and Safety, Center for Innovations in Quality, Houston, TX, USA; 3 Department of Medicine, Dell Medical School at the University of Texas at Austin, Austin, TX, USA; 4 3989 Geriatrics and Palliative Care, Baylor College of Medicine, Houston, TX, USA

**Keywords:** dementia, palliative care, hospitalization, caregiving, care coordination

## Abstract

Palliative care needs of hospitalized persons living with dementia (PLWD) and their family caregivers remain poorly understood. This mixed-methods study explored caregivers’ perspectives on challenges and support needs when hospitalized PLWD received specialist palliative care consultations. Thirty-two caregivers were recruited from two hospitals; 18 completed interviews and surveys, and 14 completed surveys only. Participants were racially and ethnically diverse (19% Black or African American, 34% Latino or Hispanic). Most identified as women (65%) and adult children (56%). Commonly reported challenges included managing health issues (59%), emotional distress (56%), and decision-making (47%). Thematic analysis of interviews revealed three themes: the value of palliative care in navigating end-of-life uncertainty in dementia, uncoordinated and reactive care during hospitalization, and lack of guidance for post-hospital transitions. While caregivers valued palliative care for emotional and decision-making support, findings underscore the need for earlier integration and improved coordination across hospital teams to better support families.

**What this paper adds**

• Family caregivers of hospitalized persons living with dementia found specialist palliative care to be valuable for communication, dementia education, and support with end-of-life decision-making.
• Family caregivers also revealed inconsistent coordination between palliative care and hospital teams, highlighting broader system-level gaps in care integration.

**Applications of study findings**

• Hospitals should more clearly define the role of specialist palliative care teams in dementia care, particularly in discharge planning and caregiver support.
• Research and policy should prioritize improving interdisciplinary coordination and embedding caregiver-centered approaches into hospital-based dementia and palliative care delivery.

## Introduction

Improving access to palliative care for 6.9 million persons living with dementia in the United States is critical (Alzheimer’s Association, 2024; Chan et al., 2023; Hanson et al., 2019). Dementia, a chronic and progressive condition, is increasingly recognized as terminal, underscoring the importance of palliative care throughout the disease trajectory (van der Steen et al., 2014). Expert recommendations call for holistic, family-centered palliative care for PLWD that support varied and evolving goals, which may include prolonging life, ensuring comfort, maintaining function, preserving personhood, and assisting family caregivers (Nishimura et al., 2024; van der Steen et al., 2014).

Hospitals are key sites for expanding access for PLWDs. Specialist palliative care is available in over 1,700 U.S. hospitals with over 50 beds, representing 83% of such institutions (Center to Advance Palliative Care, 2022) and PLWDs are 1.5 to 2 times more likely to be hospitalized than peers without dementia (Alzheimer’s Association, 2024). Evidence shows specialist palliative care can improve communication, facilitates advance care planning, and increases hospice use (Ahluwalia et al., 2018; Center to Advance Palliative Care, 2022). However, research on hospital-based palliative care for PLWDs remains limited and often excludes caregiver perspectives, relying instead on provider reports or administrative data (Courtright et al., 2020; Ding et al., 2020; Mo et al., 2021; Sharda et al., 2020). Given caregivers’ essential roles in care planning and management, and decision-making, understanding their perspectives is critical. This study explored caregivers’ views on challenges and support needs during PLWD’s hospitalization to inform strategies for advancing hospital-based palliative care.

## Methods

A qualitatively driven mixed-methods design, guided by a pragmatic approach (Creswell & Poth, 2018), was used. Given that palliative care needs of PLWDs are relatively unknown from caregivers’ perspectives, semi-structured interviews served as the primary method to elicit caregiver perspectives, complemented by a survey assessing demographics, caregiving background, and support needs. See Supplemental Material for the consolidated criteria for reporting qualitative research (COREQ) (Tong et al., 2007).

### Setting and Sample

The study was conducted in two hospitals in a U.S. city: a teaching safety net hospital with 67% of patients uninsured or underinsured, and a community hospital that is the largest medical and surgical acute care center in its area, with 55% of patients uninsured or underinsured. Purposive sampling was used to recruit adult family caregivers of PLWD providing unpaid care for persons who were hospitalized with a diagnosis of dementia or cognitive impairment and received palliative care consultation.

### Recruitment

Between June 1, 2022, and March 31, 2023, palliative care specialists and a hospitalist identified and referred eligible caregivers. Research assistants obtained informed consent and offered participants the option to complete a survey and interview or the survey alone. Participants received a $40 gift card for both survey and interview or $20 for survey-only participation (for caregivers who requested the latter option, citing reasons such as stress and time constraints from having their relative hospitalized).

### Data Collection

A semi-structured interview guide focused on what caregivers were most concerned about the relative’s care and if they received enough support in making care decisions for their relative (See Supplemental Material for the interview guide). Interviews took between 30 minutes and 60 minutes. The survey included closed-ended, Likert-scale, and open-ended items assessing: PLWD and caregiver demographic characteristics; the PLWD’s need for help with activities of daily living (ADLs) and instrumental activities of daily living (IADLs), rated from 1 (no help needed) to 4 (cannot do at all); number of recent falls, hospitalizations, and pneumonia episodes; and caregivers’ emotional, physical, and financial stress levels on a 5-point scale (1 = not at all stressful to 5 = very stressful). Caregivers checked any challenges faced from a pre-selected list (e.g., managing health issues, emotional difficulties, and decision-making), and areas where they needed more help during hospitalization (e.g., selecting long-term services and caregiving at home).

### Data Analysis

The qualitative interviews were recorded, transcribed and imported into Dedoose online software for data organization and qualitative analysis. The first author developed an initial codebook based on the literature on challenges in dementia caregiving and decision-making. Primary coding categories included the PLWD’s clinical needs; caregiver role and challenges; the specialist palliative care and primary care team roles; and types of support needed. Inductive coding allowed additional themes to emerge (see Supplemental Material for code list). After the 12th interview, no new concepts emerged. Three team members then independently coded each transcript. Team discussions resolved discrepancies and ensured reliability, with coder agreement of at least 80%. The research team then mapped the codes as emergent themes. For closed-ended survey items describing the background of PLWD and caregivers and the challenges faced, we conducted descriptive statistical analyses with SPSS/WIN 28.0. Using triangulation, we interpreted survey findings on caregiver challenges and needs alongside qualitative themes that added depth and contextual understanding.

Throughout our analysis, we considered our diverse cultural backgrounds. Our team included an East Asian female gerontologist, a White female palliative care physician and scientist from Texas, a White female research assistant from Texas, a Southeast Asian male hospitalist, and a Black research assistant of Ghanaian origin. We examined our interpretations of the data to ensure multiple perspectives were represented. Validation included the engagement of all coders in analysis and the maintenance of a comprehensive audit trail by the principal investigator for all data files, field notes, and coding memos, aligned with the standards set forth by Creswell and Poth (2018).

### Ethics

The study protocol was reviewed and approved by The University of Texas at Austin Institutional Review Board (STUDY0000261; 4/21/2022). Informed consent was obtained from all participants.

## Results

### Participants’ Characteristics

Of 86 caregivers who met eligibility from chart review, 50 were referred to the research team. Some declined to participate or could not be reached. Of the 32 caregivers who participated, 18 completed both the qualitative interview and the survey, and 14 completed only the survey. No statistically significant differences were found between those who completed only the survey and those who completed both, in terms of PLWD’s demographic characteristics, ADL, or IADL (Table 1).

**Table 1.** Characteristics of Persons Living with Dementia and Caregivers (*N* = 32 Dyads)

Characteristics	*PLWD*	*Caregiver*
Age (years), mean ± *SD*	80.78 ± 9.3	59.4 ± 13.03
Race or ethnicity,^a^ *n* (%)		
Black or African American	6 (18.8)	1 (3.1)
Asian, Native Hawaiian, or Pacific Islander	2 (6.3)	5 (15.6)
White	11 (34.4)	11 (34.4)
Latino or Hispanic	11 (34.4)	15 (46.9)
Gender, *n* (%)		
Female	19 (59.4)	20 (64.5)
Male	13 (40.6)	11 (35.5)
Education, *n* (%)		
Below high school	8 (25.8)	4 (12.9)
Completed high school	6 (19.4)	4 (12.9)
High school to college	8 (25.8)	10 (32.3)
College and above	9 (29)	13 (41.9)
PLWD living situation/admitted from, *n* (%)		
Home	24 (75)	
Group environment (e.g., assisted living)	5 (15.6)	
Other (e.g., skilled nursing facility and rehab)	3 (9.4)	
Caregiver’s relationship with the PLWD		
Child		18 (56.3)
Spouse of partner		7 (21.9)
Other		7 (21.9)
Caregiver’s contact with PLWD, *n* (%)		
Always		9 (36.0)
More than once a week		12 (48)
Once a week to a few times a month		3 (12)
A few times a year		1 (4)
PLWD in the last 3 months, *n* (%)		
Fell at least once or more	9 (29)	
Had pneumonia at least once or more	5 (16.1)	
Hospitalized at least once or more	14 (45.2)	
PLWD’s activities of daily (ADL) and instrumental activities of daily living (IADL)^b^		
ADL, mean ± *SD*	2.57 ± 0.94	
IADL, mean ± *SD*	3.27 ± 0.79	
Caregiver’s role in the care of PLWD		
Advocacy, *n* (%)		30 (93.8)
Monitoring symptoms, *n* (%)		31 (96.9)
Communication with providers, *n* (%)		29 (90.6)
Number of ADLs that the caregiver helps with, mean ± *SD*		2.52 ± 2.35
Number of IADLs that the caregiver helps with, mean ± *SD*		6.38 ± 4.17
Caregiving stress, *n* (%)		
Emotional stress, very stressful		32 (100)
Physical stress, very stressful		27 (84.4)
Financial stress, very stressful		19 (59.4)
Challenges during hospitalization, *n* (%)		
Managing relative’s health issues		19 (59.4)
Coping with emotional challenges		18 (56.4)
Making care decisions for the relative		15 (46.9)
Areas that caregivers need more help, *n* (%)		
Selecting and applying for long-term services and supports such as home health or long-term care facilities		19 (59.4)
Caregiving at home such as ensuring safety at home, managing challenging behaviors, and engaging in activities with the relative		16 (50)
Coping with emotional or physical stress		9 (28.1)

*Note.* PLWD = person living with dementia. Percentages may not sum to 100% due to missing data in some variables.

Percentages may not sum to 100% due to missing data in some variables.

^a^Although the Hispanic category denotes ethnicity, many Hispanic participants did not specify their race and identified only as Hispanic or Latino. Therefore, we are reporting race/ethnicity combined instead of separate race and ethnicity categories.

^b^ADL and IADL were measured on a scale from 1 (no help needed) to 4 (cannot do at all). The reported scores reflect the mean of six ADL items and the mean of 11 IADL items.

Overall, the 32 participants cared for racially and ethnically diverse PLWDs—19% Black or African American and 34% Latino or Hispanic. In the past three months, 45% had been hospitalized and 29% had fallen. Most PLWDs were admitted from home (75%) and required significant help with ADLs and IADLs, provided by their caregivers. During PLWD’s hospitalization, caregivers reported high stress and challenges in managing health issues (59.4%), emotional strain (56.4%), and decision-making (46.9%). When asked where more support was needed, most identified help with selecting and applying for long-term services (59.4%) and caregiving at home (50%).

### Challenges and Support Needs

Three qualitative themes on caregiver-identified challenges and support needs during PLWD’s hospitalization were: value of palliative care in navigating end-of-life uncertainty in dementia, uncoordinated and reactive care during hospitalization, and lack of guidance for post-hospital transitions, summarized with illustrative quotes in Table 2.

**Table 2.** Qualitative Themes on Caregiver-Identified Challenges and Support Needs During PLWD’s Hospitalization

Qualitative Theme	Caregiver-Identified Challenge or Need for Support	Illustrative Quotes
Value of palliative care in navigating end-of-life uncertainty in dementia	**Making care decisions and coping with emotional challenges:** Palliative care helped caregivers anticipate dementia progression, navigate with end-of-life decision-making while helping them prepare for changes in the PLWD’s condition	Our decision from that last meeting was to install a PEG, uh, a feeding tube through her stomach wall. And we’re giving—we’re giving that a trial... but I understand from the doctors **[and the palliative care team members]** that it’s not just that she’s refusing to eat, it’s that she really can’t because her intestines are so inflamed. *(*2023AS, Husband)
		She **[palliative care clinician]** was preparing us for that. You know, how she was going to feel. She’s going to hurt. She’s going to be in and out…I’ve never done anything with dementia to know the process of what’s going to happen. (3022AS, Daughter)
Uncoordinated and reactive care during hospitalization	**Managing relative’s health issues:** Hospital teams were often uncoordinated or reactive leading to missed opportunities for proactive support and timely pain management	Nobody was trying to come up with a plan for me. Now they’re trying to rush and throw something together. When her condition wasn’t as bad, when I brought her there, somebody should have been trying to make up a plan then, not let her condition deteriorate for 60 days and then, now, tell me there’s no options. (1822DS, Daughter)
		I’m very frustrated. ... Especially because it’s something that you can actually see ... as far as her pain management. I mean there are nights that she’s arching her back and she’s screaming... And it makes it extremely frustrating when nobody will make a decision. (0622DS, Grandson)
Lack of guidance for post-hospital transitions	**Caregiving at home and selecting and applying for long-term services and supports:** Discharge planning lacked practical help. Caregivers were unsure about what support or placement was appropriate and received little help in planning and preparing for transitions	When they try to send her home, I’m saying, “Okay. Well can you send her home with, with, um, home health? Can you send her home with a personal care attendant?” “Oh, well, no.” They’re not even willing to do that. And I’m like, “So y’all wanting to just send her home with me with no insurance, none of this stuff.” (0522AS, Daughter)
		That’s when the hospital started pressing me, saying “Okay. You’re going to, you know, have to start making ... decisions about what you’re going to do with her.” And I said, **“**Do I need to make a decision about ... long-term care? Do I need ... memory care or whatever?” And this is where they just completely ... gave me nothing. (2223AS, Brother)

#### Value of Palliative Care in Navigating End-of-Life Uncertainty in Dementia

Palliative care team members helped families understand the progressive nature of dementia and anticipate difficult decisions, such as whether to initiate or withhold life-sustaining treatments. Caregivers appreciated guidance framing decisions within the context of their loved one’s decline, enabling them to prepare emotionally and logistically for end-of-life care. This support was especially important given the uncertain, prolonged course of advanced dementia.

#### Uncoordinated and Reactive Care during Hospitalization

In contrast, caregivers described environment as reactive and poorly coordinated, highlighting systemic breakdowns in communication and clinical planning. Rather than proactive planning, families frequently encountered delayed or uncoordinated responses, particularly related to symptom management and care planning. Unmanaged pain, confusion over treatment plans, and last-minute decisions left caregivers feeling excluded and unsupported. These experiences underscored the emotional toll of navigating fragmented hospital systems while advocating for a loved one with dementia.

#### Lack of Guidance for Post-hospital Transitions

A third theme reflected gaps in preparing for discharge, particularly related to home-based caregiving and accessing long-term services and supports. Discharge planning often failed to provide clear options or practical support, leaving caregivers uncertain about the most appropriate level of care. Caregivers felt neglected in navigating these decisions, facing unclear next steps and barriers to obtaining supports, such as home health services or long-term care placement.

## Discussion

Family caregivers play a central role in managing the medical, emotional, and logistical needs of PLWDs especially during hospitalization (Alzheimer’s Association, 2024). In our study, most provided high levels of support and reported significant emotional stress. They described specialist palliative care as helpful for dementia education, goals-of-care communication, and end-of-life preparation. Caregivers appreciated clarity and anticipatory guidance by palliative care teams, which helped navigate uncertainty and make values-based decisions during acute decline. This aligns with prior studies showing that early, empathetic communication improves caregiver understanding, facilitates decision-making, and enhances satisfaction (Chan et al., 2023; Hanson et al., 2019).

However, caregivers also reported ongoing challenges, including inconsistent symptom management, fragmented communication, and limited support for navigating care transitions, highlighting unmet needs between specialties. Caregivers described delayed symptom management and reactive care planning, often linked to poor team coordination and unclear roles. Although palliative care is designed to address suffering, hospital complexity appeared to constrain its effectiveness. Caregivers noted insufficient guidance in discharge preparation and long-term care planning, including navigating home-based services and placement decisions. These gaps left many unprepared and unsupported at critical transition points.

National data show that most hospitalized patients seen by palliative care are discharged home or to skilled nursing facilities (Rogers & Dumanovsky, 2017). Systemic barriers contribute to coordination challenges: Medicare’s payment system does not provide sufficient reimbursement for hospital palliative care team services, requiring hospitals to absorb costs for interdisciplinary team members, while palliative care teams are frequently under-resourced and understaffed. These structural issues may be particularly pronounced given the high proportion of uninsured/underinsured patients (67% and 55%, respectively) at our study sites.

While race and ethnicity did not explicitly emerge as themes in our participants’ responses, the broader literature documents well-established racial and ethnic disparities in palliative care access and quality, including bias in pain assessment and management, which may have influenced some participants’ experiences (Bazargan & Bazargan-Hejazi, 2021). The intersectionality of race and ethnicity and socioeconomic status at our study sites may have influenced participants’ experiences with hospital-based palliative care through factors such as medical mistrust, communication barriers, and structural inequities, even when not explicitly articulated (Bazargan & Bazargan-Hejazi, 2021; Kwak et al., 2014).

Better integration across palliative care, primary care, and case management is needed to align services with families’ evolving needs and ensure continuity. Given limited specialist palliative care resources, a generalist approach by primary team—focused on early dementia education, caregiver support, and advance care planning—may be especially valuable earlier in the dementia trajectory (Weisbrod, 2022). In more advanced stages, specialist palliative care becomes essential for values-based discussions, offering anticipatory guidance, and revisiting care goals (Hanson et al., 2019).

These findings also underscore the need to reexamine how hospital-based palliative care is structured and delivered—including its scope, timing, referral criteria, and team coordination—to better serve PLWDs and their families (Mo et al., 2021). While interventions targeting burdensome symptoms in advanced dementia offer significant patient benefit, current care often lacks systematic symptom assessment (Hines et al., 2011). Our findings support the need for stronger practice guidelines, systematic pain assessment and treatment, and improved palliative care access for PLWDs. Given that fewer than 15% of hospitalized PLWDs in the United States receive palliative care (Xie et al., 2025), policy initiatives promoting systematic identification and referral processes are critically needed. Priorities for future research and practice include building dementia-specific competencies, clarifying roles, embedding caregiver education in workflows, and strengthening discharge planning and cross-team communication.

This study provides insights into PLWD caregivers’ experiences, which have been understudied, yet there are several limitations. The small sample from two hospitals in a single geographic area limits generalizability. However, the study aimed to explore, not generalize, caregiver perspectives, and included caregivers of PLWD at various stages. Of the 32 participants, 18 completed interviews and surveys; others cited stress and time constraints. Future research should include more diverse caregivers and adopt flexible, caregiver-centered approaches to improve participation among those with high caregiving demands.

Specialist palliative care provides essential communication and end-of-life support for caregivers of PLWD. Yet, gaps remain in care coordination and transition planning. Addressing these issues will require clearer team roles, stronger interdisciplinary collaboration, and more strategic use of palliative care resources.

## Supplemental Material

Supplemental Material - Family Caregivers’ Perspectives on Challenges and Support Needs in Hospital-Based Palliative Care for Persons Living With Dementia Supplemental Material for Family Caregivers’ Perspectives on Challenges and Support Needs in Hospital-Based Palliative Care for Persons Living With Dementia by Jung Kwak, Anita Chary, Sarah Stayer, Kwaku Duah Oppong, Sumin Yoon, Snehal Patel, and Elizabeth A. Kvale in Journal of Applied Gerontology.

## Data Availability

Data supporting the findings of this study are available from the corresponding author upon reasonable request.*
