# Supplementary material for: Family Caregivers’ Perspectives on Challenges and Support Needs in Hospital-Based Palliative Care for Persons Living With Dementia
Source: J Appl Gerontol. 2025 Nov 17;45(9):1696–702. doi: 10.1177/07334648251389300 (PMC13437996; doi:10.1177/07334648251389300)
Supplement: Supplementary file 1 [file sj-pdf-1-jag-10.1177_07334648251389300.pdf]

Supplemental Material 1. COREQ (COnsolidated criteria for REporting Qualitative research) Checklist

Topic	Item No.	Guide Questions/ Description	Author Responses
**Domain 1: Research team and reflexivity**			
Personal Characteristics			
Interviewer/facilitator	1	Which author/s conducted the interview or focus group?	Interviews were conducted by two research assistants.
Credentials	2	What were the researcher's credentials? E.g. PhD, MD	Both were master's students in training for social work.
Occupation	3	What was their occupation at the time of the study?	Research Assistants
Gender	4	Was the researcher male or female?	Both women.
Experience and training	5	What experience or training did the researcher have?	Both received extensive training in qualitative interviewing in healthcare settings from JK who holds formal doctoral training in Aging Studies. Both research assistants had prior experience conducting in-depth clinical interviews for migrant populations.
Relationship with participants			
Relationship established	6	Was a relationship established prior to study commencement?	Interviewers had no relationship with interviewees prior to the study.
Participant knowledge of the interviewer	7	What did the participants know about the researcher? e.g. personal goals, reasons for doing the research	Participants were informed that the interviewers were part of a research team studying the needs of hospitalized persons living with dementia (PLWD) receiving palliative care support and the experiences of caregivers and PLWD during hospitalization and discharge.

Interviewer characteristics	8	What characteristics were reported about the inter-viewer/facilitator? e.g. Bias, assumptions, reasons and interests in the research topic	One RA had interest working with older adults; the other RA was interested in working with Latino populations.
**Domain 2: Study design**			
Theoretical framework			
Methodological orientation and Theory	9	What methodological orientation was stated to underpin the study? e.g. grounded theory, discourse analysis, ethnography, phenomenology, content analysis	Deductive and Inductive Content Analysis
Sampling	10	How were participants selected? e.g. purposive, convenience, consecutive, snowball	Graduate research assistants in social work and hospitalists used electronic medical records in two urban hospitals in central Texas to identify English or Spanish-speaking family members aged 18 years or older who provided unpaid care to a PLWD who had a diagnosis of dementia or cognitive impairment and were referred to palliative care consult. Care-partners with cognitive, speech, or hearing impairments were excluded from the study.
Method of approach	11	How were participants approached? e.g. face-to-face, telephone, mail, email	Face-to-face or Telephone.
Sample size	12	How many participants were in the study?	32 caregivers participated in the overall study with 32 completing the survey and 18 of them participated in the qualitative interviews.

Non-participation	13	How many people refused to participate or dropped out? Reasons?	14 individuals did not participate due to lack of time to complete the qualitative interviews or because they could not be reached by the RA.
<u>Setting</u>			
Setting of data collection	14	Where was the data collected? e.g. home, clinic, workplace	Data collection occurred in-person in patient rooms at the hospital, through phone, or zoom.
Presence of non-participants	15	Was anyone else present besides the participants and researchers?	No
Description of sample	16	What are the important characteristics of the sample? e.g. demographic data, date	Interviews were conducted from June 1, 2022 – March 31, 2023. Participant demographics are reported in Table 1.
<u>Data collection</u>			
Interview guide	17	Were questions, prompts, guides provided by the authors? Was it pilot tested?	An interview guide with questions and prompts was developed and pilot tested within the research team. The interview guide is provided as a supplement (Supplemental Material 2).
Repeat interviews	18	Were repeat interviews carried out? If yes, how many?	For the current study, we only report qualitative interview findings from one interview. However, for the larger parent study, participants were invited to participate in the longitudinal interviews, care partners were interviewed at three different time points: first during PLWD's hospitalization and second and third time after discharge. Each interview covered a different topic, catering to the changing experiences of care partners over time.

Audio/visual recording	19	Did the research use audio or visual recording to collect the data?	Audio recording.
Field notes	20	Were field notes made during and/or after the interview or focus group?	Interviewers took notes on paper during the interview. These notes were shared with the research team.
Duration	21	What was the duration of the interviews or focus group?	30-60 minutes interviews.
Data saturation	22	Was data saturation discussed?	KJ used Excel spreadsheet software to develop an initial coding scheme on the transcripts and observed for data saturation by recording emerging codes in consecutive interviews. We achieved data saturation by 12 interviews.
Transcripts returned	23	Were transcripts returned to participants for comment and/or corrections?	No
**Domain 3: analysis and findings**			
Data analysis			
Number of data coders	24	How many data coders coded the data?	4 (First author and 3 graduate research assistants)
Description of the coding tree	25	Did authors provide a description of the coding tree?	Yes (Supplemental Material 3).
Derivation of themes	26	Were themes identified in advance or derived from the data?	Themes were derived from the data using an iterative, deductive and inductive approach.
Software	27	What software, if applicable, was used to manage the data?	Dedoose online software (https://www.dedoose.com/)
Participant checking	28	Did participants provide feedback on the findings?	No

Reporting			
Quotations presented	29	Were participant quotations presented to illustrate the themes/findings? Was each quotation identified? e.g. participant number	Quotations are identified with participant number in Table 2.
Data and findings consistent	30	Was there consistency between the data presented and the findings?	We held serial group discussions to resolve any discrepancies between the coders. This allowed coders to maintain inter-coder reliability rate of 80% and ensured consistency between presented data and themes described in the manuscript.
Clarity of major themes	31	Were major themes clearly presented in the findings?	Major themes are presented in the results section and labeled with subheadings.
Clarity of minor themes	32	Is there a description of diverse cases or discussion of minor themes?	The range of meanings associated with each theme is presented.

Developed from: Tong A, Sainsbury P, Craig J. Consolidated criteria for reporting qualitative research (COREQ): a 32-item checklist for interviews and focus groups. *International Journal for Quality in Health Care*. 2007. Volume 19, Number 6: pp. 349 – 357

Supplemental Material 2. Qualitative Interview Questions

I have a set of questions that will loosely guide our conversation, but ultimately, I'm interested in what you think is important information to share. You are the expert, and I'm excited to learn from you.

I also wanted to let you know that I'll be **recording our conversation** today. This will allow me to stay present with the conversation, and have a record of all the insights and information you share. This will be stored on a password protected website, and will not include your name or personal information. Only research team members will have access to this audio recording.

I would like to ask you about what you are concerned about regarding your relative's care.

1. What are you most concerned about with regard to your relative's care now?
2. In your opinion, what is the most important decision that you feel that has to be made now about your relative?
3. Regarding this decision, what are the options (or choices) that are available to your relative?
4. As you are considering these options you mentioned,
    a. Do you feel sure about the best choice for your relative?
    b. Do you know the pros and cons of each option?
    c. Are you clear about which pros and cons matter most to your relative?
    d. Do you have enough support and advice to make a choice?
5. Do you have any questions for the palliative care team? – any support?
6. Do you have any questions for the hospital care team?
7. Is there anything else that you'd like to share about your relative's experience during the hospital stay?

That is the end of the interview. Thank you so much for your time and sharing.

Supplemental Material 3. List of primary and secondary codes

Primary Category	Sub-Category
Caregiver role or tasks	Current tasks
Caregiver role or tasks	Future tasks (or to do)
Caregiver role or tasks	Other
Caregiver role or tasks	Past tasks (or did)
Caregiver role or tasks	Typical tasks (that caregiver does)
Caregiving Challenges	Caregiver Stress/Burden
Caregiving Challenges	Conflicts with other family member in caregiving or decision-making
Caregiving Challenges	Going Home
Caregiving Challenges	Guardianship or MPOA related
Caregiving Challenges	Patient Care
Caregiving Challenges	Placement in LTC Facility
Caregiving Challenges	Social determinants of health related for the caregiver
Caregiving Challenges	Social determinants of health related for the patient
Caregiving Challenges	Other
CG Decision-Making: Most Concerned about	Discharge planning (location)
CG Decision-Making: Most Important Decision Before Discharge	Discharge planning (location)
CG Decision-Making: Most Concerned about	Patient health or condition
CG Decision-Making: Most Important Decision Before Discharge	Patient health or condition
CG Decision-Making: Most Concerned about	Other
CG Decision-Making: Most Important Decision Before Discharge	Other
Decision-support need	Areas that the caregiver needs the most support for decision-making
General support need	Decision-making / consultation
General support need	Education/Training
General support need	Information (General or Palliative Care Team)
General support need	Other Formal Support
Decision-support need	Whose support that matter the most to the caregiver in decision-making
Healthcare provider role (Palliative care)	Communication about patient care
Healthcare provider role (General)	Communication about patient care
Healthcare provider role (Other)	Other
Satisfaction with the healthcare provider	General providers

Satisfaction with the healthcare provider	Palliative care provider
General support need	Social Support (e.g., family or friend, i.e., non formal professional support)
Other	Family obligations
Other	Looking for services/resources
Other	Managing finances
Other	Not enough time to prepare
Other	Past caregiving experience
Other	Patient care context - Past
Other	Patient Concern/Preference
Other	Strained relationship/ conflict with PLWD - Past
Other	Travel burden
